# Alginate-Based Hydrogels and Scaffolds for Biomedical Applications

**DOI:** 10.3390/md21030177

**Published:** 2023-03-13

**Authors:** Simonida Lj. Tomić, Marija M. Babić Radić, Jovana S. Vuković, Vuk V. Filipović, Jasmina Nikodinovic-Runic, Marija Vukomanović

**Affiliations:** 1 University of Belgrade, Faculty of Technology and Metallurgy, Karnegijeva 4, 11000 Belgrade, Serbia; 2 University of Belgrade, Institute of Molecular Genetics and Genetic Engineering, Vojvode Stepe 444a, 11000 Belgrade, Serbia; 3 Advanced Materials Department, Jožef Stefan Institute, Jamova Cesta 39, 1000 Ljubljana, Slovenia

**Keywords:** alginate, hydrogels, scaffolds, biomedical applications

## Abstract

Alginate is a natural polymer of marine origin and, due to its exceptional properties, has great importance as an essential component for the preparation of hydrogels and scaffolds for biomedical applications. The design of biologically interactive hydrogels and scaffolds with advanced, expected and required properties are one of the key issues for successful outcomes in the healing of injured tissues. This review paper presents the multifunctional biomedical applications of alginate-based hydrogels and scaffolds in selected areas, highlighting the key effect of alginate and its influence on the essential properties of the selected biomedical applications. The first part covers scientific achievements for alginate in dermal tissue regeneration, drug delivery systems, cancer treatment, and antimicrobials. The second part is dedicated to our scientific results obtained for the research opus of hydrogel materials for scaffolds based on alginate in synergy with different materials (polymers and bioactive agents). Alginate has proved to be an exceptional polymer for combining with other naturally occurring and synthetic polymers, as well as loading bioactive therapeutic agents to achieve dermal, controlled drug delivery, cancer treatment, and antimicrobial purposes. Our research was based on combinations of alginate with gelatin, 2-hydroxyethyl methacrylate, apatite, graphene oxide and iron(III) oxide, as well as curcumin and resveratrol as bioactive agents. Important features of the prepared scaffolds, such as morphology, porosity, absorption capacity, hydrophilicity, mechanical properties, in vitro degradation, and in vitro and in vivo biocompatibility, have shown favorable properties for the aforementioned applications, and alginate has been an important link in achieving these properties. Alginate, as a component of these systems, proved to be an indispensable factor and played an excellent “role” in the optimal adjustment of the tested properties. This study provides valuable data and information for researchers and demonstrates the importance of the role of alginate as a biomaterial in the design of hydrogels and scaffolds that are powerful medical “tools” for biomedical applications.

## 1. Introduction

The dynamics of modern life imply a variety of activities, and, therefore, a great exchange and contacts of people, which inevitably leads to the improvement of the process of living, and the development of science at unprecedented levels as an integral part of global development. Therefore, the fields of biomedical engineering and regenerative medicine are making great strides by introducing new technological approaches and strategies, as well as new biomaterials as imperatives in improving the treatment of diseases in various fields of medicine [1,2]. Biomedical engineering and regenerative medicine have a mission to bring about a personalized treatment approach that should achieve successful results in all medical fields [1,2]. The regeneration strategy of biomedical engineering should be understood as the possibility of realizing a biomimetic 3D cellular microenvironment (artificial extracellular matrix (ECM) or scaffold) that controls and directs local tissue regeneration, mainly created from a combination of natural and/or synthetic polymers, biomolecules and cells [3,4]. Natural and synthetic polymers applied in biomedical engineering include alginate, gelatin, chitosan, agarose, collagen, hyaluronic acid, carrageenan, poly(lactide-glycolide)s, and poly(hydroxybutyrate-co-valerate) [5,6,7,8,9]. Depending on the type of application, hydrogels and scaffolds should possess specific physicochemical properties (hydrophilicity, porosity, morphology, mechanical properties, biodegradability, biocompatibility) to mimic the cell environment in vivo as closely as possible [10,11,12,13,14,15,16]. Scaffolds are designed as drug delivery platforms for the controlled release of therapeutic bioactive agents (growth factors, vitamins, hormones, antibiotics) that support tissue regeneration and promote the healing process. The ability to adjust physicochemical characteristics such as hydrophilicity, porosity, morphology, mechanical properties, biodegradability, and biocompatibility (by crosslinking, blending, copolymerization, and functionalization) allows the release dynamics to be tailored to the requirements of the application [10,11,12,13,14,15,16].

**Scheme 1:**
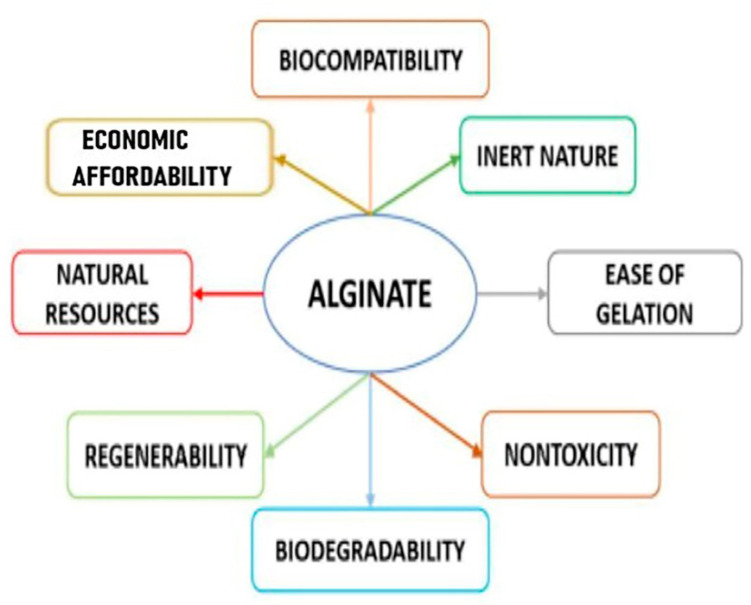
Illustration of outstanding properties of alginate.

Alginate, as a versatile polymer, has great significance due to its natural resources, suitable biocompatibility, simplicity of handling, nontoxicity, mild gelation properties and economic affordability (Scheme 1) [17,18,19,20,21,22,23,24,25,26,27,28,29,30]. The structural mimicry of an alginate hydrogel to the extracellular matrix of living tissues allows for a wide range of medical applications in dermatology and soft and hard tissue regeneration [17,18,19,20,21,22,23,24,25,26,27,28,29,30]. Alginate is a linear copolymer composed of 1,4-linked β-D-mannuronic acid (M) and 1,4-linked α-L-guluronic acid (G), with domains composed of one unit or the other (consecutive M block/residues, consecutive G block/residues) or domains with monomers in an alternating sequence (alternating MG block/residues) [17,18,19,20,21,22,23,24,25,26,27,28,29,30]. Natural, marine sources of alginate are brown algae [17,18,19,20,21,22,23,24,25,26,27,28,29,30], which belong to the *Phaeophyceae* (seaweeds) family or that are extracted from bacteria, such as *Pseudomonas* or *Azotobacter* [17,18,19,20,21,22,23,24,25,26,27,28,29,30]. Alginate is found in different forms with nonbiomedical- or biomedical-grade quality [30], low, medium, or high viscosity [30], and low or high molecular weight [30]. Alginate is hydrophilic and water-soluble, thickening in neutral conditions, and forms a hydrogel in the presence of polyvalent cations [17,18,19,20,21,22,23,24,25,26,27,28,29,30]. As a natural polysaccharide, alginate shows a pH-dependent anionic character and interacts with cationic polyelectrolytes, making it a convenient system for the loading of cells [17,18,19,20,21,22,23,24,25,26,27,28,29,30]. Although alginate may contain different endotoxin contents depending on its level of purity, its relative stability concerning biocompatibility has been proved, supporting its suitable use as a medically safe biomaterial [30].

**Scheme 2:**
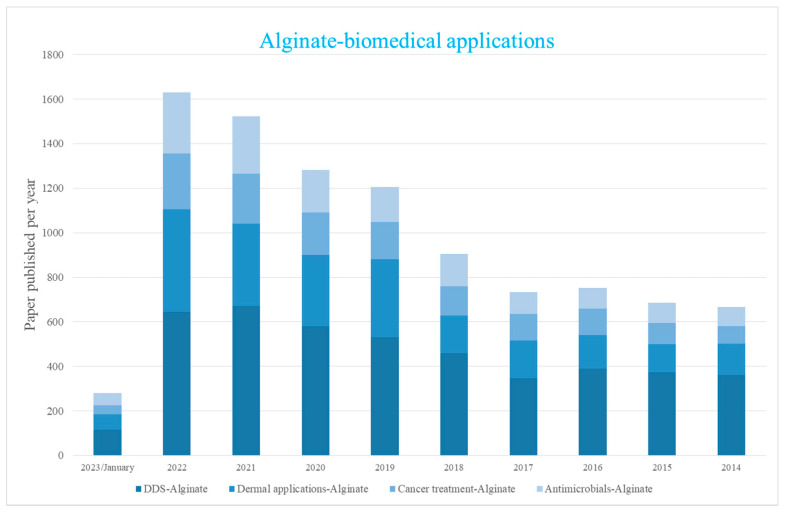
Research progress on alginate–based materials for selected fields in biomedical applications (SCOPUS database source).

Extensive research has been conducted using alginate-based hydrogels and scaffolds as therapeutic platforms for dermal applications, controlled-release systems for bioactive agents, cancer treatments, and antimicrobials. Such studies are based on continuous scientific progress, leading to new systems that are tuned to heal damaged and injured tissues at a highly personalized level using modern therapy, the action of external stimuli and the functionalization of alginate-based systems (Scheme 2). Scheme 2 shows that the last decade has seen exponential growth in research into alginate-based materials and their applications—dermal uses, drug delivery systems, cancer treatment, and antimicrobial materials. From the trend of papers published in these areas up to the end of January 2023, it can be predicted that research in the mentioned areas will continue at an advanced pace.

This review paper aims to present, as one entirety of selected areas of alginate application, its use in dermal applications, controlled-release systems, cancer treatment and antimicrobial materials and the most significant achievements in these areas. For each research area, emphasis was placed on the composition of hydrogels and scaffolds, as well as the special influence of alginate on the most important tested properties for selected biomedical applications. In one place, the reader can briefly read valuable scientific information and be informed about scientific achievements in the mentioned areas of biomedical applications of alginate.

## 2. Alginate-Based Hydrogels and Scaffolds for Dermal Application

Bioengineered dermal polymeric scaffolds play an important role in the treatment of full-thickness skin disorders [31]. Whether it is a case of surface skin injuries or acute wounds and more complex, chronic wounds (diabetic and venous ulcers), the approach for the efficient regeneration and restoration of the skin requires multidisciplinarity, emerging from the knowledge of engineering and biomedical sciences. The process of healing comprises overlapping stages: hemostasis, inflammation, proliferation and remodeling/maturation of the skin tissue. It is orchestrated by a series of interconnected molecular and cell reactions, all involved in the physiological metabolism of the living organism. Regarding the complexity of the tissue regeneration and the restoration of the optimal skin parameters, the timeline is often uncertain, especially when microbial infection of the damaged tissue occurs. The healing is prolonged, and the therapy needs to be enriched with a strong antibacterial character.

Bioactive, antibacterial and pro-regenerative dermal scaffolds present a class of bioengineered, advanced, multiscale platforms in regenerative medicine [32,33]. These biomaterials are designed to transcend the limitations of the traditional treatment methods, enabling the restoration of the biological functions of the skin through the application of bioinspired-designed scaffolds with the powerful synergistic effect of a polymeric 3D architecture and carefully incorporated bioactive agents and cells. Their unique property is the ability to recreate a physiological environment similar to the natural ECM and to promote cell growth and vascularization [34]. Dermal scaffolds are classified as synthetic, natural and hybrid (natural-synthetic), based on the nature of the polymeric constituents used for the fabrication [35]. Although synthetic monomers and polymers used for hydrogel and scaffold components provide excellent mechanical strength and the possibility to tailor the microstructure and its porosity, current trends in biomedical engineering are leaning towards natural compounds. Thus, the use of biological polymers, such as polysaccharides and proteins, becomes dominant when it comes to the design of dermal scaffolds.

As a natural polysaccharide with extraordinary properties for biomedical applications, alginate is one of the most dominant constituents of bioactive dermal scaffolds [36]. Due to specific biological properties, alginate enhances the treatment of injured skin tissue and prevents bacterial infection through its inherent antibacterial activity [37]. In regenerative medicine, alginate and its derivatives have been used in various formulations such as hydrogels, scaffolds, films, microspheres, and fibers [26]. Due to its hydrophilicity, water solubility and specific functional groups, alginate is easily blended with other biopolymers (collagen, hyaluronic acid, chondroitin sulfate, fibrin, fibronectin, agarose, chitosan and silk) to form hydrogels applicable as wound healing biomaterials [38].

Alginate (Alg) as a main component is combined with biopolymers such as coagulating and antibacterial agent fibrinogen (Fib), nisin (antibacterial agent, Nis), and ethylenediaminetetraacetic acid (antibacterial agent, EDTA) for wound healing applications [39]. The alginate solution used for the hydrogel preparation was 4.0% (*w*/*v*). The concentration of alginate in hydrogels was constant (0.87 mM), as was that of fibrinogen (7.17 µM) and EDTA (50 mM), while the concentration of nisin was varied (0.03, 0.08, and 0.15 mM). The interaction between fibrinogen and alginate was studied using spectroscopic techniques to confirm the use of fibrinogen in the hydrogel dressing. The results showed that the fibrinogen conformation was slightly affected by the alginate presence and was a suitable option for the preparation of the dressing. Further, the Alg-Fib@Nis-EDTA hydrogel was prepared and characterized. Properties of the hydrogel such as the formation of desired bonds, antibacterial resistance, swelling, porosity, mechanical resistance, optimal release rate, acceleration of the blood clotting process, and cell cytotoxicity were determined. The designed scaffold absorbed the secretions of the wound environment due to its high swelling capacity (about 2000%). Gradually released nisin and EDTA inhibited the growth of both Gram-positive and Gram-negative bacterial species. It exhibited no toxicity to the human epidermal cell line and also showed a very favorable function in accelerating blood clotting. Based on the in vivo results, the prepared hydrogel showed adequate antibacterial activity accompanied by suitable therapeutic efficacy in chronic wounds in terms of improving the rate of epithelialization and collagen formation processes [39].

Multifunctional self-healing hydrogels have been synthesized using alginate functionalized with aldehyde, polyetherimide, and strontium ions to promote angiogenesis and accelerate tissue regeneration [40]. Hydrogels were prepared by mixing a solution of sodium alginate aldehyde (10%) with a solution of polyetherimide (10%) (ratio 1:1) and then doping with strontium ions. Continuous release of Sr^2+^ ions was achieved by oxidized alginate, which continuously affected the growth of vascular endothelial cells. The antibacterial properties of the prepared alginate-based materials prevented infections and ensured a beneficial healing process.

Combining alginate and chitosan with medical agents of natural origin, Aloe vera extract and honey, efficient hydrogels were prepared for the treatment of injured skin tissue [41]. The hydrogels were prepared using a solution of sodium alginate 2% (*w*/*v*) and chitosan (2.00, 1.60, 1.95, 1.55% (*w*/*v*)). The beneficial properties of each used component for the hydrogel designs contributed to the beneficial properties of the synthesized hydrogels as constructs. The results showed that the designed hydrogels possessed a porous structure with interconnected pores, the size of which was suitable for cell adhesion, proliferation and migration. Additionally, their physical and structural characteristics were favorable for wound healing. The presence of Aloe vera in the hydrogels generally improved the special application of the hydrogel for wound healing. The synergistic effects of the used components on biocompatibility, hemolytic activity, cell attachment and prevention of bacterial infections caused by *Staphylococcus aureus* (inhibitory zone 23 mm) and *Pseudomonas aeruginosa* (14 mm) were demonstrated. According to the results of this study, the use of hydrogels containing honey and Aloe vera based on alginate and chitosan polymers led to the formation of appropriate structures and biocompatibility for use in the treatment of damaged tissue, especially wounds.

The microgel-in-gel concept was applied to regulate and tune the hydrogel degradation rate and controlled-release properties. Alginate hydrogels are useful in the treatment of diabetic wounds [42]. However, the limited adaptability of the ion crosslinking method prevents delicate manipulation of physical properties in response to different wound conditions. This issue was solved by using microgel particles (made of zinc ions and coordinated through a complex of carboxymethyl chitosan and aldehyde hyaluronic acid) as a crosslinker. A cation was then introduced as a second crosslinker to create a double polymeric network. The method enabled precise regulation of the hydrogel’s character, including the rate of biodegradation and controlled drug release. The optimized hydrogels enabled the infiltration of cells in vitro and promoted the regeneration of diabetic wound tissues in vivo. The results showed that the addition of microgel as a novel crosslinker created flexibility during alginate hydrogel preparation, adapting to different applications during diabetic wound therapy. Microgels allowed for more chemical modulations and resulted in much greater adaptability. Sodium alginate solution (1%, 2 mL) was mixed with different weights of microgels (0.5, 1 and 2 mg). The microgel-in-gel design provided a precise release rate of fluorescein isothiocyanate-labeled bovine serum albumin (FITC-BSA), compared to cation-only crosslinked sodium alginate hydrogels that exhibited a less desirable “splash” effect. In this way, microgels were used to control the rate of drug release and avoid the initial rapid drug release. These types of obtained hydrogels have been used as physical support for 3D cell culture in vitro, allowing cells to infiltrate the scaffold and ultimately improve epithelization and closure of the diabetic wound.

Sodium alginate/gelatin (SA/Ge) scaffolds were crosslinked with calcium ions and loaded with cellulose nanocrystal (CNC) to advance their physicochemical properties [43]. Alginate/gelatin/cellulose nanocrystal (SA/Ge/CNC) scaffolds were obtained through the electrostatic interaction of sodium alginate and gelatin, sodium alginate crosslinked with calcium ions, and embedding of CNC. The scaffolds were prepared from alginate and gelatin solutions (3 wt%), in different ratios, and then loaded with cellulose crystals. The SA/Ge and SA/Ge/CNC scaffolds were tested by scanning electron microscopy, swellingstudy, tensile strengths and contact angles. The involvement of CNC produced hybrid SA/Ge/CNC scaffolds with the desired porous network, moderate swelling behavior, and superior mechanical strength (the range of 18 to 45 MPa). An in vitro cytotoxicity and cell growth assay using mouse embryonic fibroblast cells validated that the SA/Ge/CNC scaffold was non-toxic and prompted cell adhesion and proliferation. The in vivo skin regeneration experiments using the SA/Ge/CNC scaffold group showed an improved skin wound healing process with accelerated re-epithelialization, increased collagen deposition and faster extracellular matrix remodeling. Based on the morphological, swelling and mechanical properties, the optimal ratio of sodium alginate/gelatin was found to be 2/1. The increase in gelatin content indicated a non-uniform structure with larger pores, which ultimately led to the lower mechanical strength of the hydrogel, implying the beneficial effect of alginate on the morphological and mechanical features of the hydrogels. The obtained results indicated that SA/Ge/CNC hybrid scaffolds with improved mechanical performance and wound healing efficiency are biomaterials favorable for the regeneration of skin defects.

The hypothesis that the dense layer protects the wound surface and maintains a moist environment on the wound surface was the basis of research [44]. The appropriate formulation of bioink hydrogel (sodium alginate/gelatin/collagen) was developed to simulate the physiological structure of the skin using 3D printing. The proportion of hydrogel corresponding to each layer was optimized. Specific variations of the components corresponding to the layers of the skin were designed. Sodium alginate was crosslinked with calcium ions and provided stability and shape fidelity of the scaffold. On the other hand, the biological inertness of alginate limited the adhesion and migration of cells, which was compensated with the introduction of gelatin and collagen. These results revealed that the scaffolds had interconnected macroscopic channels. Sodium alginate/gelatin/collagen scaffolds reduced wound skin contraction and accelerated wound healing and re-epithelialization in vivo. It has been confirmed that this is a fast and economical way to produce skin scaffolds intended for clinical application.

Skin-mimicking constructs for the treatment of skin wounds were created using three-dimensional (3D) printing technology [45]. A bilayer membrane (BLM) scaffold was designed and printed, consisting of an outer poly(lactic-co-glycolic acid) (PLGA) membrane and a lower alginate hydrogel layer, which mimicked the epidermis and dermis of the skin. PLGA (Mw 70–88 kDa, LA/GA = 50/50) and analytical-grade sodium alginate were used. The multi-porous alginate hydrogel in the BLM scaffold promoted cell adhesion and proliferation in vitro, while the PLGA membrane prevented the bacterial attack and retained the moisture content of the hydrogel. Skin regeneration using a bilayer scaffold was compared with the regeneration of PLGA, alginate hydrogel and an untreated defect in vivo. Tissue samples were analyzed by histopathological and immunohistochemical CD31 staining. mRNA expression levels of collagen markers (collagen type 1 alpha 1 (COL1a1) and collagen type 3 alpha 1 (COL3a1)) and inflammatory markers (interleukin-1β (IL-1β) as well as tumor necrosis factor (TNF-α)) were determined. The application of the BLM scaffold resulted in the highest levels of skin regeneration by increasing neovascularization and increasing collagen I/III deposition. Three-dimensionally printed BLM scaffolds have improved wound healing and have proved suitable for a wide range of wound dressing applications or skin substitutes.

To obtain wound dressings with good, moist, healing, and biocompatibility properties, hydrogels based on sodium alginate and gelatin were prepared [46]. The ratios of gelatin to alginate were 70/30, 60/40, 50/50, 40/60, and 30/70, respectively, which affected the properties of the hydrogel. The samples with higher alginate content had structures with uniform sodium alginate matrices and gelatin as a dispersed phase in a droplet-like formation. The swelling capacity of the hydrogels was decreased as the sodium alginate content increased, which was attributed to the morphology of the sodium alginate matrices with smaller pores and their influence on the diffusion of water molecules. The maximum water content of all swelled hydrogels was in the range of 74–94%. The increasing concentration of sodium alginate affected the viscoelastic properties of the hydrogels, in terms of increasing the values of storage and loss modulus.

Poly(vinyl alcohol)-sodium alginate (PVA-SA) hydrogel wound dressing membranes loaded with sodium ampicillin have been developed using the physical crosslinking method [47]. Different fractions of PVA and SA (0, 25, 33, 50, 65, and 75%) solutions were prepared. The physical entanglement between PVA and SA was verified by IR analysis. The results showed that the incorporation of SA in the physically crosslinked PVA network significantly affected its molecular structure and morphological features. The surface morphology and roughness of PVA membranes were strongly influenced by SA content. The swelling ability of the hydrogels rose (from 1500% to 4200%) with the increase in sodium alginate content (from 0% to 75%). The release rate of ampicillin was decreased (from 50% to 38%) with the increase in sodium alginate content (from 0% to 75%), probably due to the interactions between functional groups of alginate and ampicillin. The prepared hydrogels exhibited a high capability of protein adsorption, high hydrolytic degradation, and appropriate antimicrobial activity. Therefore, ampicillin-loaded PVA-SA hydrogels have been shown as favorable wound dressing materials with acceptable blood compatibility through hemocompatibility results.

Gelatin (Gel), sodium alginate (SA), and hyaluronic acid (HA) based series hydrogels for biomedical applications were prepared by a freeze-drying method using 1-ethyl-3-(3-dimethyl aminopropyl) carbodiimide (EDC) as a crosslinker (ratio gel/SA/HA = 1/8/1, 3/6/1, 4.5/4.5/1, 6/3/1 and 8/1/1) [48]. The results showed that the Gel/SA/HA hydrogels were successfully crosslinked by the crosslinking agent. All Gel/SA/HA hydrogels with different compositions had highly homogeneous and interconnected pores. The compositions had no significant effect on the surface and cross-sectional morphologies of the Gel/SA/HA hydrogels. The incorporation of sodium alginate enhanced the water vapor transmission capacity of the hydrogels. Gelatin had a low hydrophilic behavior, while sodium alginate exhibited a relatively high hydrophilic behavior. The results indicated that the Gel/SA/HA hydrogel cross-linked via EDC was a wound dressing material capable of the adequate provision of a moist environment for comfortable wound healing.

The influence of alginate content (1, 2, 3 and 4% *w*/*v*) on the properties of alginate-chitosan hydrogels as wound dressing materials was evaluated [49]. The increase in alginate concentrations led to a decrease in the swelling capacity of the hydrogels, but the holding capacity was increased up to 86%. The MTT test on L929 fibroblast cells showed that the investigated hydrogels exhibited biocompatibility up to grade for wound dressing applications.

The development of alginate-based hydrogels and scaffolds for applications in dermal tissue regeneration and the treatment of various skin diseases is proceeding at an accelerated pace (Table 1). Based on previous research, it is expected that alginate will be one of the first choices for the production of new bioengineering polymeric hydrogels and scaffolds. Future innovations should combine advanced technology and scientific knowledge to replicate important physicochemical properties of human skin tissue and thus facilitate tissue restoration. Tailored alginate-based dermal scaffolds with highly tunable biocompatibility, mechanical properties, biodegradability, and controlled-release performance with embedded materials of human origin (placenta, amniotic fluid and other types of tissue and cells) are expected to be the focus of future scientific research.

## 3. Alginate-Based Hydrogels and Scaffolds in Drug Delivery Systems

Alginate is a commonly used polymer of natural origin for the design and fabrication of drug delivery systems (DDS) due to its well-known advantageous properties [1,2,3,4,5]. The presence of active functional (carboxylic and hydroxyl) groups allows a wide range of possibilities for biochemical, chemical, or enzymatic modification necessary for the design of various functional and effective DDSs. The design of a DDS containing alginate as a single/combined polymer requires the control of important properties such as porosity, mechanical strength, swelling behavior, degradation, and cell and/or bioactive agent loading and release properties according to the desired drug release profile (sustained/controlled), drug administration route (oral, transdermal, parenteral, pulmonary, localized/targeted) as well as to the challenges that must be overcome by bioactive molecules. Therefore, controlled or sustained release of different bioactive agents such as quercetin, ciprofloxacin, and rifampicin from alginate-based hydrogels has been investigated [50,51,52,53,54]. Chitosan-alginate hydrogels were prepared and tested to achieve simultaneous and sustained release of ciprofloxacin, amoxicillin and vancomycin for individual and combined therapy. Hydrogels were synthesized by complexation between polysaccharide chitosan chains (20 mg/mL) and alginic acid sodium salt from brown algae (10 mg/mL) followed by crosslinking with Ca^2+^ ions to make the hydrogels more stable and to enhance functionality. Drugs were loaded into the hydrogel network by an in situ loading method, as single-loaded and drug combination-loaded formulations where the ratio between the total polysaccharide content (alginic acid and chitosan) and antibiotic was defined as 20/1 (weight ratio, mg). The morphology, swelling capacity, antibacterial activity, cytotoxicity and release of all formulations were investigated. Obtained results showed that all of hydrogels possessed a porous structure with no homogenous pore size and distributions due to the presence of the loaded drugs. Encapsulation efficiency was in the range of 91–95% for single-loaded and 87–95% for drug combination-loaded formulations; equilibrium swelling was reached within 60 min for all hydrogels, with a maximum swelling at pH of 7.40 and lowest at pH of 4.00. Obtained release data showed an initial “burst” release for all investigated DDSs, followed by a sustained release for 10 h, where more than 80% of the drug was released, independently from the pH of the media and the type of loading (individual or multiple), making the hydrogels appropriate for DDS applications for individual and combination therapies [55]. pH-responsive alginate-based hydrogels as oral DDSs, especially for colon-targeted systems, were created by free radical copolymerization using 3-[(methacryloyl amino)propyl trimethyl ammonium chloride] (MAPTA), methacrylic acid (MA), 2-hydroxyethyl methacrylate (HEMA) and sodium alginate from brown algae (Carl Roth). The content of sodium alginate was the same in all samples (5 g, 2% *w*/*w*), while the contents of MAPTA, MA and crosslinker MBA were varied. To evaluate the hydrogels as DDSs, diclofenac sodium was used as a model drug, and the achieved amount of loaded drug was 22.8%. In vitro drug release study was performed in simulated intestinal (pH of 7.00) and gastric (pH of 1.20) fluid, suggesting limited drug release at pH of 1.20 (4.5% for 3 h) due to deficient ionization of carboxylic groups and controlled release at pH of 7.00 for 20 h (95%). The presented results classified the investigated hydrogels with a 1/1/1 monomer ratio for a pH-sensitive DDS for oral applications [56]. A diclofenac sodium sustained-delivery system was developed based on hydrogels of different concentrations of sodium alginate and 2-acrylamide-2-methyl propane sulfonic acid, where the amount of alginate was varied from 0.25 g/100 g to 0.75 g/100 g. The hydrogel with the highest content of alginate (0.75 g/100 g) had the highest swelling capacity and showed the highest drug loading efficiency (above 98%, 98.97 mg of drug/450 mg dry gel). An in vitro drug release study was performed at different pH values, confirming pH-controlled release, where 99% of diclofenac was released at a pH of 7.40, while 20% of the drug was released at a pH of 1.20. Release data showed that the diclofenac release rate was increased by increasing alginate content in the hydrogels and highlighted the capability of the hydrogels for sustained delivery of diclofenac sodium by adjusting alginate and crosslinker concentrations [57]. Curcumin- and graphene oxide-incorporated hydrogels based on alginate (2.0% *w*/*v*) suitable for use as patches for the local treatment of squamous cell carcinoma were developed. Firstly, curcumin was loaded onto graphene oxide nanosheets and then blended into a hydrogel based on alginate crosslinked by Ca^2+^. The results showed that loading of 2.5% and 5% curcumin enhanced the biocompatibility of the hydrogels, while higher amounts did not induce any effect. Curcumin-loaded hydrogels containing GO showed sustained curcumin release (50% after 96 h) due to the affinity of GO for curcumin, resulting in π−π stacking interactions between the aromatic rings of the polyphenols and sp^2^ carbon layer of the carbon nanostructure and a strong cytotoxic effect on squamous cell carcinoma, which made these hydrogels advantageous DDSs for long-term anticancer therapy [58]. Injectable porous reduction-responsive hydrogels based on alginate were obtained by inverse electron demand Diels–Alder reaction between the alginate-norbornene (Alg-Nb) group and a water-soluble poly(ethylene glycol) (PEG), which is an attractive “click” reaction for hydrogels based on polymers of natural origin. The content of alginate was the same in all samples (Alg-Nb 2% *w*/*v*), while different concentrations of the crosslinker were used. The obtained hydrogels exhibited higher swelling capacity, porous morphology, drug-loading efficiency of 92% and suitable mechanical properties. It was observed that the drug-loading efficiency of the hydrogels increased by increasing the concentration of the crosslinker and that the ionic interaction between carboxylate groups of alginate and amine groups in the drug also increased drug loading. The in vitro doxorubicin release was stimulated by the presence of glutathione (GSH), and release results showed higher doxorubicin release (93%) in the presence of GSH compared to release in PBS solution without GSH (36%) after 11 days. Additionally, the doxorubicin-loaded hydrogels induced cytotoxicity in cancer cells, promoting the hydrogels as candidates for stimuli-sensitive DDSs [59]. In recent decades, the popularity of alginate-based scaffolds in biomedical applications has been growing exponentially. Among their excellent cell proliferation and osteoblast differentiation strengths, alginate-based scaffolds also possess a capability for controlled delivery of different types of bioactive agents for tissue regeneration applications. Karimi et al. investigated the in vivo regenerative effects of a daphne mucronate (DM)-loaded alginate scaffold (1.25 g alginate in 100 mL distilled water) for wound healing purposes. They found that alginate-based scaffolds loaded with 1.25 g DM/100 g alginate achieved controlled release of daphne mucronate, as a rich source of herbal antioxidants, and accelerated the tissue regeneration process. The daphne mucronate-loaded alginate-based scaffolds prompted fast expression and high levels of EGF, bFGF and MMP13 genes essential for the wound regeneration process and substituted pathological tissues with normal, physiological forms [60]. An alginate dialdehyde-gelatin hydrogel reinforced by lysozyme and silica-calcium nanoparticles and loaded by cerium was designed to obtain hydrogel scaffolds for bone tissue engineering. Scaffolds containing 5% alginate were used. Obtained in vitro results showed that the hydrogel scaffolds improved proliferation, adhesion and differentiation of pre-osteoblast cells due to the excellent biocompatibility of alginate dialdehyde-gelatin hydrogel, the bioactivity of the cerium and silica-calcium nanoparticles, and the stabilizing effect of lysozyme on the scaffold structure. Additionally, the scaffolds provided a release of lysozyme throughout the 21-day immersion time and decreased the proliferation of MG63 osteosarcoma cells due to the effect of lysozyme. All obtained results supported the convenience of the scaffolds for bone tissue engineering able to induce bone regeneration, address infection and inhibit tumor formation [61]. Silk fibroin/sodium alginate (SF/SA, SF (4%) and SA (1%)) hydrogel scaffolds loaded by teicoplanin (TEC) and phenamil (PM) for treating chronic osteomyelitis were investigated. The results of an in vitro release study of the scaffolds indicated the sustained and pH-sensitive release of TEC and PM with a higher release rate in alkaline pH for 10 days. The release of PM from the scaffolds at pHs of 5.50, 7.40, and 8.50 was about 54, 56, and 59% after 40 days, respectively. The results indicated that the scaffold constructs diminished the “burst” release of PM due to polymeric chains of the hydrogel acting as a barrier for PM and prolonging the release. The release of TEC from the scaffolds at three different pHs was about 83, 90 and 89% for 35 days. It was observed that TEC was released in a sustained and pH-dependent manner from the SF/SA scaffolds. The scaffolds promoted cell viability, ALP activity and mineralization of the matrix as well as antibacterial activity for 35 days at different pHs. The best in vivo results were obtained by treating methicillin-resistant *Staphylococcus aureus* (MRSA) infected rat bone with scaffolds loaded with TEC, which implied that the scaffolds were favorable for a dual-drug delivery system in the treatment of chronic bone infection [62].

Based on previous scientific research, alginate was a very convenient component in drug delivery systems for the release of a variety of lower and higher molecular weight drugs as well as drugs with low bioavailability and poor solubility in water (Table 2). This is explained by its safety, biocompatibility, biodegradability, nontoxicity, unique structure, physicochemical properties, and simple methods of preparation as well as a level of therapeutic activity that is significant for the modern design of various drug delivery systems. An important property of alginate is its ability to form different structures in aqueous solutions such as hydrogels and 3D porous scaffolds that open up the possibility of designing a variety of pharmaceutical formulations including “smart”, target-oriented formulations to overcome the serious side effects of drugs. The intelligently guided design of advanced multifunctional drug delivery systems based on alginates is a modern strategy for personalized medical/pharmaceutical approaches and provides effective and complete healing in a shorter time interval with better disease management.

## 4. Alginate-Based Hydrogels and Scaffolds for Cancer Treatment

In recent decades, biodegradable alginate-based biomaterials have shown significant potential for the design of drug delivery systems (DDSs) for anticancer drugs [63,64]. Alginate is an ideal material for designing and forming multifunctional DDSs for cancer imaging and therapy due to its advantages such as biocompatibility, biodegradability, and convenient modification to different derivates with flexible properties, structure, functions and applications [65]. Moreover, it has the specific ability to form hydrogels with a high water content that makes them similar to native soft tissue. The alginate-based hybrid system of cisplatin-loaded alginate nanogel encapsulated in an injectable alginate hydrogel crosslinked with calcium ions for the treatment of ovarian cancer was developed [66]. To form the cisplatin-loaded alginate nanogel, cisplatin (6 mg/mL) was mixed with an aqueous solution of alginate (2.0 mg/mL), heated at 95 °C, dialyzed and lyophilized. Then, the cisplatin-loaded alginate nanogel was redispersed in a CaCl_2_ solution and added to an alginate solution (50 mg/5 mL). The obtained system enabled high cisplatin loading capacity and the sustained release of cisplatin for over a week and showed significant in vivo antitumor activity. This therapy presented a modern approach to the treatment of advanced-stage ovarian cancer with KRAS mutations [67]. A hybrid system based on alginate (100 mg/50 mL) and graphene oxide (GO) for DDS application was synthesized with calcium ions (0.1 wt%) as the crosslinker, followed by freeze-drying procedure. The obtained DDS was evaluated for loading and controlled release of methotrexate (MTX), which is used as one of the chemotherapeutic agents for cancer therapy. The release study confirmed the pH- and electro-responsive release of MTX due to the pH-sensitive properties of alginate and the conductive ability of GO. It was observed that the cumulative release of MTX was 14% at pH 7.40 and 9% at pH 6.00, but electrical stimuli increased the release of MTX to 29% and 37% at −0.2 V and −0.4 V, respectively. These findings represented a good starting point for the synthesis of smart DDSs based on polymers of natural origin [68]. Some therapies involve the use of several therapeutic agents. Wu et al. investigated hydrogels with two co-assembling domains for structural reinforcement of the hydrogel and the controlled release of two drugs, cisplatin (CDDP) and irinotecan (IRN). It was observed that a fast release of CDDP occurred before the sustained release of IRN, suggesting that the release profile depended on the fraction ratio of IRN-loaded alginate nanoparticles in nanomaterials. Obtained in vitro release profiles of CDDP showed that 45% of the drug was released for 9 h, suggesting a fast release of CDDP where release rates of CDDP were unaffected by the amounts of INR-loaded alginate nanoparticles. On the other hand, the cumulative release of IRN was slower (11.3% for 9 h) and sustained (6 days 60.7%) with tunable properties by varying the fraction of IRN-loaded alginate nanoparticles in the nanosystem (from 0.4 wt% to 0.6 wt%). The obtained results provided a generalized strategy to realize the differential release of multiple active agents in dual therapy [69]. In addition to the wide use of alginate in the preparation of single/combined DDSs, its application for the design of 3D scaffolds as tumor models is also significant [69,70]. Its suitability for various biomedical applications implies that alginate has great potential in cancer therapy and research in this field. As can be seen throughout this review, alginate is not only an excellent candidate for the design of smart and stimuli-responsive DDSs for anticancer active agents but also an efficient 3D scaffolding material for cell cultures. Alginate-based hydrogels have been confirmed to improve traditional anticancer therapies and overcome existing disadvantages such as systemic toxicity and tumor resistance. Summarized data for alginate-based hydrogels and scaffolds for cancer treatment are presented in Table 3.

## 5. Alginate-Based Hydrogels and Scaffolds as Antimicrobials

Antimicrobial materials are a very important class of materials in the biomedical area, especially now in the era when new types of microbes appear and pose a huge threat to the human race. Therefore, the choice of material types, in combination with bioactive agents, as well as production technologies are the focus of today’s modern scientific research. Some materials possess inherent antimicrobial properties, and some must “be enriched” with antimicrobial agents to achieve the final function. Alginate-based materials have a significant impact in the field of antimicrobial materials. This particularly applies to the treatment of skin function disorders. Wounds infected with a pathogenic microorganism usually become chronic and lead to the progression of life-threatening inflammatory diseases, and may also lead to cancer. Pathogenic microorganisms resistant to antimicrobial agents are an increasingly pressing problem that threatens the successful treatment of various types of infections [71]. Heavy metals, copper, zinc, gold and silver have been used for more than a millennium due to their known favorable antimicrobial properties. An advanced approach in terms of local delivery of heavy metals by choosing an appropriate drug release system is the answer to the threat of microbial infections [71]. Alginate, as the basic polymer component in infection treatment systems, has proved to be the first choice. Hydrogel beads were obtained using alginate (0.5% *w*/*v*) with 1,3:2:4-di(4-acyl hydrazide)-benzylidene sorbitol (0.3% *w*/*v*), a component that self-assembles to form a gel [72]. The antimicrobial properties of the hydrogel beads were acquired by the loading of silver ions and in situ reduction to form silver nanoparticles. Gels showed good antimicrobial properties against the drug-resistant bacterium *Pseudomonas aeruginosa* (PA14) and Vancomycin-resistant *Enterococcus faecium* (VAR). Calcium alginate greatly improves the thermal stability and mechanical properties of the gel. The combination of natural and synthetic materials allowed the hydrogel to have suitable mechanical features and the ability to manage the silver nanoparticle size and hydrogel shape. Silver nanoparticles, obtained similarly and stabilized by polyvinylpyrrolidone, were loaded in hydrogel made using alginate (2% *w*/*v*) and collagen (type I, 1% *w*/*v*) [73]. Inclusion of alginate allowed for high water absorption capacity of the hydrogel, as well as improved mechanical properties. The charged surface of two natural polymers allowed for the formation of a polyelectrolyte complex. Hydrogel material displayed low cytotoxicity, with distinct antimicrobial properties against *Staphylococcus aureus* and *Escherichia coli*, dependent on the concentration of silver nanoparticles. Antimicrobial scaffolds were also created by combining negatively charged alginate (3% *w*/*v*) with positively charged polysaccharide chitosan (2.5% *w*/*v*) to form a polyelectrolyte complex network and loading it with silver nanoparticles [74]. The cryogelation method afforded a porous thin-layer membrane with a large surface area that was tested for use as an air-filtration membrane to efficiently purify the air of microbes. Hydrogels showed inhibitory activity against *S. aureus* and *E. coli*. Novel antibacterial 3D-printed scaffolding materials obtained from a combination of alginate (5% *w*/*v*) and crystalline nanocellulose (CNC, 3% *w*/*v*) with incorporated silver nanoparticles have been reported [75]. CNC is a biocompatible polymer of natural origin with a crystal structure and high surface area that is virtually non-toxic, making it a great component for the creation of biomaterials for medical applications. Combining it with alginate afforded the ability to greatly improve the mechanical properties of the hydrogel, with samples resisting breakage until a force of 3.58 N was applied. Hydrogels loaded with 10 and 100 µg/mL of silver nanoparticles displayed antimicrobial properties against *S. aureus* and *P. aeruginosa*, while not exhibiting any cytotoxic effects. Contrasted with the abovementioned hydrogels and scaffolds, materials based on alginate modified with a 3-(trimethoxy silyl)propyl)-octadecyl dimethyl ammonium chloride to produce quaternary ammonium salts have shown antimicrobial activity by themselves, even without the incorporation of silver [76]. An aqueous solution of (3-(trimethoxysilyl)propyl)-octadecyl dimethyl ammonium chloride (0.02 M) was added to sodium alginate aqueous solution (4% *w*/*v*), and the resulting solution was added dropwise to a solution of calcium chloride (5% *w*/*v*). Loading the quaternary ammonium salt-based hydrogel with silver ions greatly increased its inhibitory activity against a wide variety of pathogenic microorganisms, including *Candida albicans*, Methicillin-resistant *Staphylococcus aureus* (MRSA), *S. aureus*, *E. coli* and *P. aeruginosa* (ATCC^®^10145 and ATCC^®^27853), compared to conventional alginate-based hydrogels. The modified alginate structure also allowed the slow and constant release of silver ions, effectively reducing their toxicity while retaining antibacterial effects, making it a beneficial wound dressing material.

The antimicrobial properties of silver nanoparticles depend on the level of particle aggregation. Aggregated particles have a lower ratio of surface area to volume, leading to the non-optimal release of silver ions and reducing their potency to inhibit pathogenic microorganisms [77]. Functionalized Ag nanoparticles with thioctic acid were prevented from aggregating to a large extent, and their antimicrobial properties were improved when incorporated into alginate hydrogel [77]. An aqueous solution of sodium alginate (1.5% *v*/*v*) was used for the production of hydrogels. The addition of alginate enabled the formation of a stable hydrogel, with uniform distribution of silver nanoparticles. Modified silver particles were used to crosslink calcium alginate by ionic interactions between carboxyl functional groups and Ca^2+^ ions, which allowed equal distribution of Ag particles in hydrogels.

Besides silver, other heavy metals with antimicrobial properties were also loaded in alginate hydrogels. Malagurski et al. managed to synthesize microbeads by crosslinking the alginate polymer mixture in a solution of copper(II) ions [78]. By varying the concentration of the Cu^2+^ ion solution in the range of 13.5–270 mM, added to the 1.9% *w*/*v* solution of sodium alginate, different sizes of microbeads were obtained. The Cu^2+^ ion content controlled the release rate, with higher concentrations showing an initial “burst” effect, and lower Cu^2+^ content leading to slower ion release from the hydrogel beads. Microbeads loaded with 100 µmol/g Cu^2+^ showed rapid antibacterial effects against *E. coli* and *S. aureus*. The same authors also incorporated mineralized zinc (phosphate and carbonate) in alginate (1.9% *w*/*v*) by electrostatic extrusion, using zinc(II) ions gelling solution [79]. Mineralized zinc was obtained by adding a 100 mM gelling solution of zinc nitrate to a saturated aqueous solution of sodium carbonate or sodium hydrogen phosphate. Hydrogels with mineralized zinc showed higher zinc(II) content and produced a sustained release of ions, as well as better stability in the physiological medium concerning non-mineralized samples. Both mineralized and non-mineralized samples showed strong antimicrobial effects against *S. aureus* and *C. albicans*, while concerning *E. coli*, the effect depended on the type of sample. Three-dimensionally printed antimicrobial alginate/bacterial cellulose-based materials, with incorporated copper nanoparticles, active against *E. coli* and *S. aureus* strains were prepared [80]. Alginate content was varied from 1 to 4% *w*/*v*. Two ionic crosslinking methods were tested, one using calcium ions with their subsequent replacement with copper ions by ion exchange and another crosslinking method with copper ions only. Copper nitrate aqueous solution (2.5 mg/mL) was treated with sodium borohydride solution to produce copper nanoparticles that were incorporated into alginate hydrogels. The ion exchange method yielded more-stable materials. Alginate is often used for 3D printing and to fabricate printable inks. New 3D printing inks based on antimicrobial alginate/bacterial-cellulose hydrogel structures with increased alginate content (4% *w*/*v*) showed improved printability compared to pure alginate hydrogels [80], and the addition of copper provided antimicrobial effects against *E. coli* and *S. aureus* strains.

Antimicrobial cerium ion/chitosan crosslinked alginate (1% *w*/*v*) biopolymer films were produced using formulations of Ce^3+^ and Ce^3+^/chitosan (1% *w*/*v*) solutions as a crosslinker to compare results between films crosslinked using Ca^2+^ and Ca^2+^/chitosan solutions [81]. Crosslinking of alginate was performed either by di- and trivalent cations or interactions with positively charged polyelectrolyte chitosan. Ce^3+^ and Ce^3+^/chitosan crosslinked films displayed better mechanical features (elastic modulus of 40.3 kPa and elongation on a break of 26.1%) compared to calcium-crosslinked alginate hydrogels (elastic modulus of 3.51 kPa and elongation on a break of 20.2%), and antibacterial properties. Furthermore, Ce^3+^/chitosan film was shown to have the best antibacterial properties against *E. coli* and *S. aureus* among the samples and was used as a flexible, ultraviolet-protecting, antibacterial wound dressing. The injectable iron(III) ions-crosslinked alginate-hyaluronic acid hydrogel was produced as a hydrogel with typical shear-thinning properties [82]. Alginate content was 3% *w*/*v,* and hyaluronic acid content was 0.75% *w*/*v*. Ionic crosslinking was performed by simply mixing the alginate-hyaluronic acid mixture with a Fe^3+^/EDTA complex solution (0.1 mol/L Fe^3+^ and 0.05 mol/L EDTA). The hydrogel was injected by application of shear stress, and it self-healed within seconds after the removal of shear, due to reversible and dynamic metal–ligand interactions between ferric ions and carboxyl groups of alginate-hyaluronic acid polymers. Besides good biocompatibility and degradability (complete degradation within 7 days), sustained ferric ions release from these hydrogels resulted in high antimicrobial activities against several types of bacteria, such as Gram-negative *E. coli* and Gram-positive *S. aureus*, as well as oral pathogenic bacteria *Streptococcus mutans* and *Porphyromonas gingivalis*.

Sodium alginate-based films were used to investigate the antimicrobial and antifungal activities of nine kinds of encapsulated essential oils [83]. Films were formed by adding different volumes (0.1, 0.5 and 1.0 mL) of essential oils to an aqueous solution of sodium alginate (3% *w*/*v*) and glycerol (1% *v*/*v*). Dynamic mechanical analysis revealed that the addition of Igepal CO-520 surfactant improved the dispersion of the oils in the alginate matrix but resulted also in a lowering of Young’s modulus of the films (6 to 1 GPa), as well as their elongation rate at the brake, but to a lesser extent. By monitoring the release of oil, it was found that oils released from the films inhibited bacterial and fungal growth depending on the type and concentration of the oil. Almost all oils, except for chamomile blue, inhibit the growth of *C. albicans*. Out of nine tested oils, only cinnamon, lemongrass and peppermint oil inhibited the growth of *E. coli*. The antibacterial activity of lignin model dehydrogenated polymer (DHP) incorporated in alginate hydrogel was studied by using different bacterial strains and bacterial biofilms [84]. DHP in DMSO (1% *w*/*v*) was added to an aqueous solution of sodium alginate (~2.1% *w*/*v*), and CaCl_2_ (0.5% *w*/*v*) solution was used to crosslink the alginate. In addition to bacterial strains that were cultured in laboratory conditions, wild strains isolated from patients’ wounds were studied as well. The results showed that the lignin model dehydrogenated polymer was active against all tested Gram-positive and Gram-negative bacterial strains and had no cytotoxic effect on human Hep2c and HCjE cells. The best results were obtained against *L. monocytogenes*, *P. aeruginosa*, *S. typhimurium* and *S. aureus*. Although alginate does not have antimicrobial activity, alginate hydrogel retained and immobilized bacteria, allowing for a reaction of the active substance with the bacteria [85]. To investigate the angiogenic potential and antibacterial activity of heparin, three different concentrations (1, 5 and 10 µg/mL, marked A, B and C, respectively) were loaded into commercially available alginate wound dressing biomaterials [85]. The loaded hydrogel samples displayed a much higher swelling rate (1524, 1202 and 1279% for samples A, B and C, respectively), compared to the non-loaded alginate sample (838%). The results indicated good absorption capability of hydrogels and increased angiogenic and antimicrobial ability caused by the addition of heparin. The best results for antibacterial activity against *E. coli* with no cytotoxic effect were obtained with the highest heparin concentration of 10 µg/mL.

Sodium alginate and PEG-based hydrogels were prepared by Diels–Alder (DA) click chemistry between furyl-modified alginate (1.5% *w*/*v* aqueous solution) and PEG by two maleimide molecules [86]. The hydrogels were subsequently chemically modified through grafting with cysteine (CYS)-terminated antimicrobial peptide HHC10–CYS in different concentrations (1, 2, 3 and 4 mg/mL) by thiol–ene reaction between the oxy-norbornene group and the thiol. The results of testing showed that hydrogels designed in this manner had good antimicrobial properties, with a sterilization rate against *E. coli* > 98.5% for all samples, and were suitable for use as coatings for implantable medical devices. The healing efficacy of sodium alginate (10% *w*/*v*) hydrogel was advanced by coupling it with different concentrations of honey (2–10%) and performing dual ionic, with calcium cations, and covalent crosslinking with maleic anhydride [87]. The dually crosslinked sodium alginate hydrogels with 4% honey concentration produced a material with appropriate stiffness (2.32 µN/nm), swelling behavior (highest swelling index of 0.6) and controlled degradation (87.36% after 12 days), providing wound dressing material with the best environment necessary to improve cell growth and proliferation. This wound dressing also had good antimicrobial potential against Methicillin-resistant *S. aureus* and *E. coli*. Alginate hydrogels, prepared from 1% *w*/*v* aqueous solution of sodium alginate crosslinked by calcium chloride, were loaded with neomycin or propolis by direct blending or by adding neomycin (0.01% neomycin sulfate solution) or propolis-loaded alginate microparticles prepared by the extrusion dripping method to alginate hydrogels [88]. The resulting films were tested for physical and antimicrobial penetration. The loading of neomycin and propolis, in both types of films, provided the samples with high swelling degrees of up to 100%. The films also provided sufficient antibacterial action to stop the penetration of microorganisms in a wound, which is essential for absorbent wound dressing materials. Summarized data for alginate-based antimicrobial materials are presented in Table 4.

## 6. Alginate, Gelatin and 2-Hydroxyethyl Methacrylate Based Hydrogel Scaffolds for Biomedical Applications

**Scheme 3:**
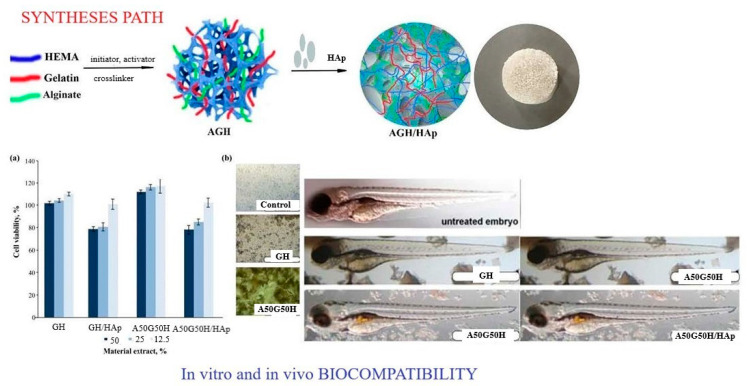
Syntheses path for preparation AGH/HAp scaffolds; in vitro (fibroblast cell line (**A**)) and in vivo (zebrafish (**B**)) biocompatibility of alginate/gelatin/2-hydroxyethyl methacrylate/apatite scaffolds.

A multifunctional polymeric system was designed using alginate combined with gelatin, 2-hydroxyethyl methacrylate and bioactive components and agents so that hydrogel scaffolds with specific properties suitable for biomedical applications were obtained. Biocompatible, porous and degradable hydrogel scaffolds made of alginate/gelatin/2-hydroxyethyl methacrylate (AGH), embedded with inorganic agent apatite (HAp), were made using the cryogelation method (Scheme 3). A balance was achieved between the biological and mechanical properties of the created hydrogel scaffolds for advanced tissue regeneration applications. The component weight ratio was gelatin/HEMA = 0.2/0.8 and alginate/gelatin/HEMA = 0.1/0.1/0.8. The sodium salt of alginic acid from brown algae was used for all syntheses. It has been shown that the loading of linear alginate chains affects the mechanical properties in terms of increasing modulus and decreasing porosity. All hydrogel scaffolds showed a completely hydrophilic character, which made them suitable biomaterials for scaffolds with improved adhesion, proliferation and differentiation of cells in the tissue regeneration process. An in vitro degradation study was conducted for 3 months, and the results showed that alginate affected the degradability of the scaffolds due to its hydrophilic character, which promotes higher degradability. An in vitro biocompatibility test on a fibroblast cell line and an in vivo study on a zebrafish model confirmed that the obtained scaffolds had satisfactory biocompatibility and that alginate contributed to better biocompatibility. These specific properties indicated that the created alginate/gelatin/2-hydroxyethyl methacrylate/HAp hydrogel scaffolds possessed advanced properties for biomedical applications [89].

**Scheme 4:**
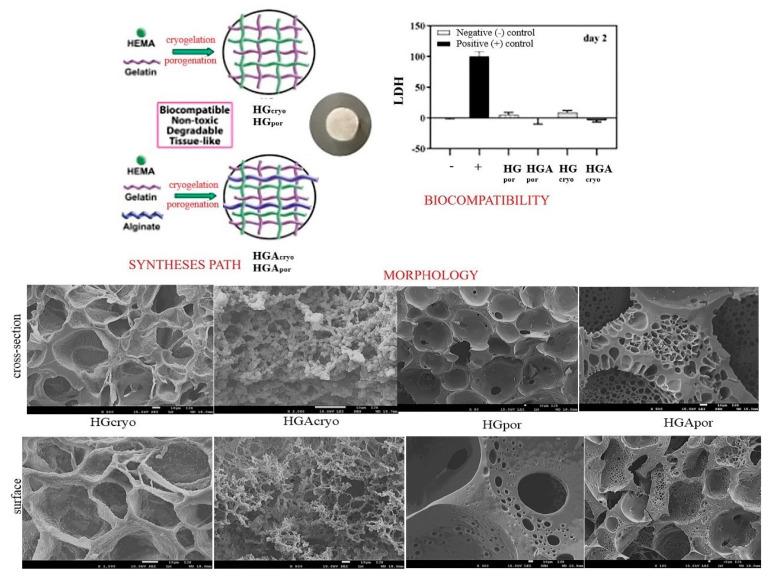
Syntheses path, biocompatibility and morphological features of IPN and semi–IPN 2-hydroxyethyl methacrylate/gelatin intertwined with alginate and doped with apatite scaffolds (the bar is 10 μm).

Bioactive 2-hydroxyethyl methacrylate (HEMA)/gelatin (G)/alginate (A) biomaterials for scaffolds in combination with inorganic agent apatite (HAp) were produced by cryogelation (−18 °C) and porogenation (using porogen agent) methods (Scheme 4). The ratio of alginate to gelatin as the weight ratio was A/G (50/50 for cryosamples and porogen samples). The sodium salt of alginic acid from brown algae was used for all syntheses. Our scientific concept was based on the creation of a “replica” of the native extracellular matrix through the design of interpenetrating networks of polymeric hydrogels. The key influence of the method of preparation and introduction of linear alginate as an interpenetrant into the HEMA/gelatin polymeric networks on the properties of the obtained hydrogel scaffold was confirmed. The morphological patterns were satisfactory interconnected pore structures depending on the interpenetrating linear alginate chains and the preparation method. Porosity values ranged from 66.38–84.25% and indicated the dominant influence of linear alginate chains, as well as scaffold preparation methods. Mechanical properties expressed by Young’s modulus were in the range of 0.99–9.75 MPa. Alginate was found to improve the mechanical features of the cryogelled scaffold and decrease the porogenated scaffold. Porosity values ranged from 66.38–84.25%. The mechanical properties given using Young’s modulus were in the range of 0.99–9.75 MPa. In vitro degradation was studied over a time interval of 16 weeks and showed a weight loss percentage of 1.59–5.04%. This behavior during degradation is explained by the higher porosity of the cryosamples and the hydrophilicity of alginate, which allows faster and easier penetration of water inside the scaffolds and a higher degree of degradation. The cell viability test showed excellent biocompatible behavior of the scaffolds. Alginate as a component improved biocompatibility. Cryogelled scaffolds have been shown to possess superior biofunctionality necessary for the tissue regeneration process—full hydrophilicity, degradability, appropriate mechanical properties, and favorable biocompatibility. The introduction of the inorganic agent apatite into the cryogelled scaffolds showed a favorable cell adhesion capacity and good biocompatible and non-toxic properties. All synthesized scaffolds performed equally in terms of metabolic activity and osteoconductivity. The study showed that cryogelled scaffolds with/without HAp were a breakthrough for improving osteoconductivity and intensifying hard tissue regeneration [90].

Embedding of iron(III) oxide in 2-hydroxyethyl methacrylate/gelatin/alginate hydrogel scaffolds resulted in iron(III) oxide/2-hydroxyethyl methacrylate/gelatin/alginate hybrid hydrogel scaffolds (multicomponent Fe/H/G/A biomaterials). A multifunctional, biocompatible, porous, and degradable hydrogel scaffold platform based on 2-hydroxyethyl methacrylate, gelatin, and alginate integrated with iron(III) oxide was obtained using a simple and effective, modified porogenation method. To achieve a balance between the biological and mechanical properties of Fe/H/G/A hydrogels and to satisfy their final biomedical application as scaffolds and efficient drug delivery system, the composition of the hydrogels was varied. The proportion of alginate was shown concerning gelatin as a weight ratio A/G (100/0, 50/50 and 70/30). Alginic acid sodium salt from brown algae was used for all syntheses. Fe/H/G/A hydrogels showed pH sensitivity with maximal swelling at pHs 2.20 and 7.40 at 37 °C as well as tunable swelling capacity by simply controlling their composition. The sample containing the highest content of alginate showed the highest swelling degree. With an increase in the alginate content in these samples, there was a decrease in porosity, because the linear alginate chains rearranged themselves within the HG network and filled the free space (porosity range of 64.97–72.38%). All Fe/H/G/A hybrids manifested fully hydrophilic character, which made them convenient for scaffolding biomaterials to support population, adhesion, and differentiation of cells. In vitro degradation studies for 3 and 6 months showed that samples containing higher alginate content had a higher level of degradability. The samples degraded up to 23.90% after 3 months, while after 6 months, they degraded up to 45.69%. In vitro biocompatibility assays confirmed that hybrid scaffolds with higher alginate content possessed excellent biocompatible behavior. The hydrophobic drug curcumin was efficiently loaded into the Fe/H/G/A hybrids, with an efficiency of over 95%. Fe/H/G/A hybrids, as controlled curcumin release platform with high release ability for hydrophobic drugs, indicated that the more alginate these hybrids contained, the better the release performances were achieved. The specific properties of the obtained iron(III) oxide/2-hydroxyethyl methacrylate/gelatin/alginate hybrids developed in this study exhibited favorable features for biomedical applications, as well as for the design of a controlled drug release platform for hydrophobic drugs [91].

Multifunctional graphene oxide/2-hydroxyethyl methacrylate/gelatin/alginate hydrogel scaffolds (GO/H/G/A) as scaffolding biomaterials were set as suitable controlled drug release systems. The hydrogels were prepared using the modified porogenation method. The ratio of alginate to gelatin as a weight ratio was A/G (100/0, 50/50 and 70/30). Alginic acid sodium salt from brown algae was used for syntheses. The engineered hydrogel scaffolds showed satisfying swelling capacity, full hydrophilicity, porosity (range of 56 to 76%), mechanical properties (Young’s modulus values from 1.69 to 4.78 MPa), and in vitro degradation up to 40% for 6 months. Alginate was a key component that influenced all of the tested properties, and with an increase in content, the tested properties were improved. The entrapment efficiency of curcumin (therapeutic bioactive agent) was above 99%. The controlled release process indicated excellent curcumin release performances for the obtained scaffolding systems, emphasizing that the sample with the highest alginate content showed the best release performances. An in vitro cytotoxicity assay on healthy human fibroblasts revealed non-toxic and favorable biocompatibility. The unique performance of GO/PHEMA/G/A hydrogel scaffolds indicated applications in various medical fields, as well as favorable controlled drug release systems [92].

**Scheme 5:**
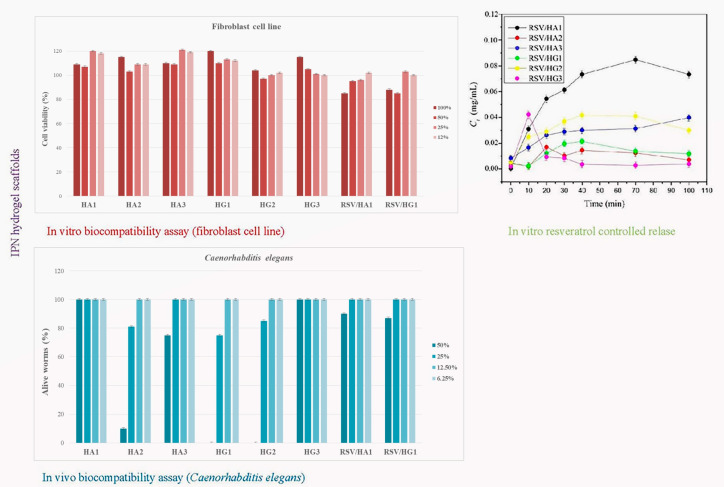
In vitro and in vivo biocompatible and controlled resveratrol release performances of IPN HA and HG scaffold series.

Interpenetrating hydrogel networks (IPN) of 2-hydroxyethyl methacrylate/alginate (HA) and 2-hydroxyethyl methacrylate/gelatin (HG) hydrogel scaffolds were obtained to design biocompatible and controlled resveratrol release platform (Scheme 5). Interpenetrating hydrogel networks consisting of 2-hydroxyethyl methacrylate (H), alginate (A), and gelatin (G) were synthesized using cryogelation at −18 °C for 24 h (ratio H/A and H/G = 0.8/0.2). Three combinations with two types of crosslinkers were used (N-ethyl-N′-(3-dimethyl aminopropyl)carbodiimide hydrochloride-EDC and N-hydroxysuccinimide-NHS). Multifunctional, bioactive scaffolding biomaterials possess advantageous absorption ability, specific morphological patterns with interconnected pores, full hydrophilicity, a porosity range of 62.36 to 85.20%, and mechanical properties expressed by Young’s modulus from 1.40 to 7.50 MPa, dependent on the scaffold’s composition and the used crosslinkers and their concentrations. In vitro and in vivo assays performed on human fibroblast cell line and the worm *Caenorhabditis elegans* exhibited favorable biocompatible features (cell viability and nematodotoxicity) of prepared IPN HA and HG hydrogel scaffolds that depended on the scaffold’s composition and the used crosslinkers and their concentrations. The release parameters of resveratrol/HA and resveratrol/HG systems were determined and suitable release models are defined. The most favorable properties for biomedical applications of these two series of IPNs were shown by samples containing alginate and EDC crosslinker. According to the authentic features, scaffolding platforms based on IPN HA and HG series were effective controlled resveratrol release systems [93].

This research opus for hydrogel scaffolds based on alginate, gelatin, HEMA and bioactive agents has shown that the introduction of alginate as a component in these polymeric multi-systems, which contained several other polymeric components and bioactive agents, had an advanced, improved effect on most of the properties that were examined and most important for biomedical applications (Table 5). It should be noted that there are different types of alginates as well as newer and more sophisticated methods of synthesis and characterization. All research efforts are aimed at creating scaffolds that are closer in properties and functions to natural tissues. The main goal is to create bionic tissues and organs that perform nearly as well as natural tissues and organs and provide a longer and better quality of life for the human population.

## 7. Conclusions and Perspectives

This review paper provided insight into selected areas of alginate-based hydrogels and scaffolds used for biomedical applications and emphasized its extraordinary properties as an outstanding biomaterial. The most attractive features of alginate include biocompatibility, mild gelation conditions, and simple modifications to prepare alginate derivatives with new and advanced properties. Alginate-based hydrogels and scaffolds have proved to be beneficial biomaterials for dermal treatment, controlled-release drug delivery systems, cancer treatments and as antimicrobials. The key elements in creating the most ideal scaffolds are the choice of components (in this case, choice of alginate type), synthesis methods, bioactive agents and their combination, which is the pinnacle of knowledge of all the skills and areas involved in biomedical engineering. Inevitably, one of the main aspirations in the next decade will be the emergence of alginate-based hydrogels and scaffolding materials that can be used clinically with promising results. Therefore, more well-designed research and clinical studies are needed to achieve this goal by elucidating the efficacy of alginate-based scaffolding biomaterials that have a significant impact in all fields of medicine. Furthering our current understanding of the fundamental properties of alginate and developing new types of tissue-interactive alginate-based hydrogels and scaffolds may validate future advances in biomedicine and regenerative medicine. The ability to engineer new types of alginates with precisely controlled chemical and physical features, unlike the limited repertoire available from natural sources, designed for a specific application could revolutionize the use of these biomaterials.

## Figures and Tables

**Table 1:** Summarized data of alginate-based hydrogels and scaffolds for applications in dermatology.

Alginate-Based Biomaterials	Applications	References
Alginate, fibrinogen/nisin, ethylenediaminetetraacetic acid hydrogels	Treatment of chronic wounds	[39]
Sodium alginate aldehyde, polyetherimide hydrogels doped with strontium ions	Wound healing, skin defect repair	[40]
Sodium alginate, chitosan, Aloe vera, honey hydrogels	Repair of damaged skin tissue, wound healing	[41]
Sodium alginate hydrogels crosslinked with carboxymethyl chitosan, aldehyde hyaluronic acid microgels coordinated by zinc ions	Treatment of diabetic wounds	[42]
Sodium alginate, gelatin hydrogel scaffolds reinforced with cellulose nanocrystal	Wound healing, skin tissue regeneration	[43]
Sodium alginate, gelatin, collagen hydrogels	Wound healing/Treatment of full-thickness skin disorders	[44]
Sodium alginate, poly(lactic-co-glycolic acid) hydrogel scaffolds	Wound healing/Treatment of full-thickness skin wounds	[45]
Sodium alginate, gelatin hydrogels	Wound healing	[46]
Sodium alginate, poly(vinyl alcohol) hydrogel membranes loaded with sodium ampicillin	Wound healing	[47]
Sodium alginate, gelatin, hyaluronic acid hydrogels	Wound healing	[48]
Sodium alginate, chitosan hydrogels	Wound healing	[49]

**Table 2:** Summarized data for alginate-based hydrogels and scaffolds applications in drug delivery systems.

Alginate-Based Biomaterials	Applications	Reference
Sodium alginate, chitosan	Controlled quercetin delivery system	[50]
Sodium alginate, coated with chitosan and Tween 80	Nanocarrier system for inhalation delivery of rifampicin	[51]
Sodium alginate, high methoxyl pectin, silver nanoparticles	Controlled ciprofloxacin delivery system	[52]
Sodium alginate, phthaloyl chitosan, poly(ethylene glycol)	Controlled ciprofloxacin delivery system	[53]
Mannose-modified alginate	Targeted antigen (ovalbumin) delivery to dendritic cells	[54]
Sodium alginate from brown algae, chitosan	Simultaneous and sustained release of ciprofloxacin, amoxicillin and vancomycin for individual and combined therapy	[55]
Sodium alginate from brown algae, methacrylic acid, 2-hydroxyethyl methacrylate, methacrylic acid and 3-[(methacryloyl amino)propyl trimethyl ammonium chloride]	Colon-targeted diclofenac sodium delivery system	[56]
Sodium alginate, 2-acrylamide-2-methyl propane sulfonic acid	Diclofenac sodium sustained delivery system	[57]
Sodium alginate, graphene oxide	Curcumin delivery system for the local treatment of squamous cell carcinoma	[58]
Alginate-norbornene, poly(ethylene glycol)	Stimuli-sensitive doxorubicin release system	[59]
Sodium alginate	Scaffolds for controlled release of daphne mucronate for wound healing application	[60]
Sodium alginate, gelatin, lysozyme, silica-calcium	Scaffolds for controlled release of cerium for bone tissue regeneration application	[61]
Sodium alginate, silk fibroin	Scaffolds for sustained and pH-sensitive release of teicoplanin and phenamil for treating chronic osteomyelitis	[62]

**Table 3:** Summarized data for alginate-based hydrogels and scaffolds for application in cancer treatment.

Alginate-Based Biomaterials	Applications	References
Sodium alginate	Doxorubicin controlled release systems for cancer therapy	[64]
Sodium alginate from brown algae	Cell encapsulation scaffolds, injectable cell delivery vehicle for cancer therapy	[65]
Sodium alginate	Cisplatin release system for ovarian cancer treatment	[66]
Sodium alginate, graphene oxide	Electro- and pH-responsive methotrexate release system for cancer therapy	[67]
Alginate nanoparticles	Controlled cisplatin and irinotecan release system	[68]
Sodium alginate, mammary epithelial cells, fibroblasts, endothelial cells	Scaffolds for anticancer therapies	[69]

**Table 4:** Summarized data for alginate-based antimicrobial materials.

Alginate-Based Biomaterials	Applications	References
Calcium alginate, 1,3:2:4-di(4-acyl hydrazide)-benzylidene sorbitol, silver	Self-assembling antimicrobial beads	[72]
Alginate, collagen, silver	Antimicrobial wound dressing	[73]
Alginate, chitosan, silver	Polyelectrolyte antimicrobial air-filtration membrane	[74]
Alginate, crystalline nanocellulose, silver	Antimicrobial ink for 3D printing	[75]
Alginate, 3-(trimethoxy silyl)propyl)-octadecyl dimethyl ammonium chloride, silver	Antimicrobial scaffolding materials	[76]
Alginate, silver with thioctic acid	Antimicrobial hydrogel	[77]
Copper alginate	Antimicrobial hydrogel	[78]
Zinc alginate	Antimicrobial hydrogel	[79]
Copper alginate, bacterial cellulose	3D-printed antimicrobial materials	[80]
Alginate, cerium ions/chitosan	Antimicrobial wound dressing	[81]
Iron(III) alginate, hyaluronic acid hydrogel	Injectable antimicrobial hydrogel	[82]
Sodium alginate, glycerol, essential oils	Wound dressing films	[83]
Alginate, lignin model dehydrogenated polymer	Wound dressing hydrogel	[84]
Alginate, heparin	Angiogenic and antimicrobial hydrogels	[85]
Furyl-modified alginate, maleimide-modified PEG	Antimicrobial hydrogels formed by Diels-Alder reaction	[86]
Calcium alginate, honey, maleic anhydride	Cutaneous wound healing	[87]
Alginate, propolis, neomycin	Dressings for diabetic ulcers	[88]

**Table 5:** Summarized data for alginate, gelatin, and HEMA based hydrogel scaffolds with bioactive agents.

Alginate-Based Biomaterials	Applications	References
Sodium alginate, gelatin, HEMA, HAp	Tissue regeneration	[89,90]
Sodium alginate, gelatin, HEMA, iron(III) oxide, curcumin	Dermal treatment, drug delivery systems, antimicrobials	[91]
Sodium alginate, gelatin, HEMA, GO, curcumin	Dermal treatment, drug delivery systems, antimicrobials	[92]
Sodium alginate, gelatin, HEMA, resveratrol	Dermal treatment, drug delivery systems, tumor treatment	[93]

## Data Availability

Not applicable.
